# 16S rRNA Gene Amplicon Sequencing of the Gut Microbiota of Chimaera phantasma (Silver Chimaera) Captured off Koshimoda in Suruga Bay, Japan

**DOI:** 10.1128/mra.01149-22

**Published:** 2023-04-03

**Authors:** Tasuku Ogita

**Affiliations:** a Department of Biomolecular Innovation, Institute for Biomedical Sciences, Interdisciplinary Cluster for Cutting Edge Research, Shinshu University, Nagano, Japan; Montana State University

## Abstract

The intestinal microbiota of Chimaera phantasma (silver chimera) (two females and one male) collected off Koshimoda in Suruga Bay in April to May 2022 were comprehensively analyzed. Bacteria belonging to the phylum *Proteobacteria* were the dominant species. Occupancy rates of other bacterial phyla differed greatly among the samples.

## ANNOUNCEMENT

Gut microbiota, which facilitate host homeostasis (1, 2), have been analyzed in many fish species (3, 4) but rarely in deep-sea fish species (5, 6). Individuals of Chimaera phantasma (silver chimera) (7) were caught in a trawl net (depth of approximately 200 to 250 m) off Koshimoda in Suruga Bay, Japan (34.86486N, 138.72713E). Sampling of their intestinal contents was performed within 48 h after capture and storage at 4°C. After laparotomy, their gastrointestinal tracts were removed, and the lower stomach contents were collected in a sterile 50-mL centrifuge tube and stored at −80°C until DNA extraction. Specimens were preserved in our laboratory by liquid immersion. In ZircoPrep minitubes (Nippon Genetics), fish gut contents (5 to 10 mg) were mixed with InhibitEX buffer (200 μL; Qiagen). The samples were shaken for 5 min (Bead Ruptor 12 homogenizer; Omni), and DNA was extracted using the QIAamp Fast DNA stool minikit (Qiagen). The 10-μL first PCR mixture contained 5 μL of 2× PCR buffer with 0.2 μL of KOD FX Neo DNA polymerase (Toyobo Co.), 0.2 μL each of 10 μM primers 341f (5′-ACACTCTTTCCCTACACGACGCTCTTCCGATCT-NNNNN-CCTACGGGNGGCWGCAG-3′) and 805r (5′-GTGACTGGAGTTCAGACGTGTGCTCTTCCGATCT-NNNNN-GACTACHVGGGTATCTAATCC-3′) for amplification of the 16S rRNA gene (V3 to V4 regions), 2 μL each of 2 mM deoxynucleoside triphosphates (dNTPs), and 1 μL of the gut content DNA. The reaction conditions were as follows: 94°C for 2 min, 30 cycles of 98°C for 10 s, 55°C for 30 s, and 68°C for 30 s, and finally 68°C for 7 min. The PCR products were purified using the AMPure XP system (Beckman Coulter). The 10-μL second PCR mixture contained 1 μL of 10× *Ex Taq* buffer, 0.1 μL of *Ex Taq* high-sensitivity (HS) DNA polymerase (TaKaRa Bio), 1 μL each of primers 2ndF (5′-AATGATACGGCGACCACCGAGATCTACAC-Index2-ACACTCTTTCCCTACACGACGC-3′) and 2ndR (5′-CAAGCAGAAGACGGCATACGAGAT-Index1-GTGACTGGAGTTCAGACGTGTG-3′) (Illumina), 2 μL each of 2.5 mM dNTPs, and 2 μL of the first-round PCR product. The indexed PCR conditions were as follows: 94°C for 2 min, 12 cycles of 94°C for 30 s, 60°C for 30 s, and 72°C for 30 s, and finally 72°C for 5 min. These PCR products were purified using the AMPure XP system and quantified using a QuantiFluor double-stranded DNA (dsDNA) system (Promega) and Synergy H1 plate reader (BioTek). A library quality check was performed using the dsDNA 915 reagent kit and a Fragment Analyzer (Advanced Analytical Technologies). The PCR products were sequenced (2 × 300-bp paired-end reads) using the MiSeq reagent kit v.3 and a MiSeq sequencer (Illumina). The reads obtained were demultiplexed and trimmed using fastx_barcode_splitter and fastx_trimmer, respectively, in FASTX-Toolkit v.0.0.14 (8). All low-quality reads (Q scores of <20) and merged reads (<130 bp) were filtered out using Sickle v.1.33 (9). The remaining paired-end reads were combined using FLASH v.1.2.11 (10). Sequence data analysis, including clustering and chimera checking, was performed using the QIIME2 pipeline v.2022.2 (https://qiime2.org). Sequences showing ≥99% similarity were grouped into operational taxonomic units (OTUs) using the SILVA database v.138 (11) (Table 1). Default parameters were used for bioinformatic analyses except where otherwise noted.

**TABLE 1:** Summary of samples analyzed in this study

Sample	Host species	Collection date (yr-mo-day)	No. of raw sequencing reads	No. of quality-filtered reads	No. of observed OTUs	DRA accession no.
C_1	Chimaera phantasma	2022-4-10	34,807	23,735	13	DRR413612
C_2	Chimaera phantasma	2022-4-17	43,573	31,845	13	DRR413613
C_3	Chimaera phantasma	2022-5-15	43,594	32,204	99	DRR413614

**FIG 1:**
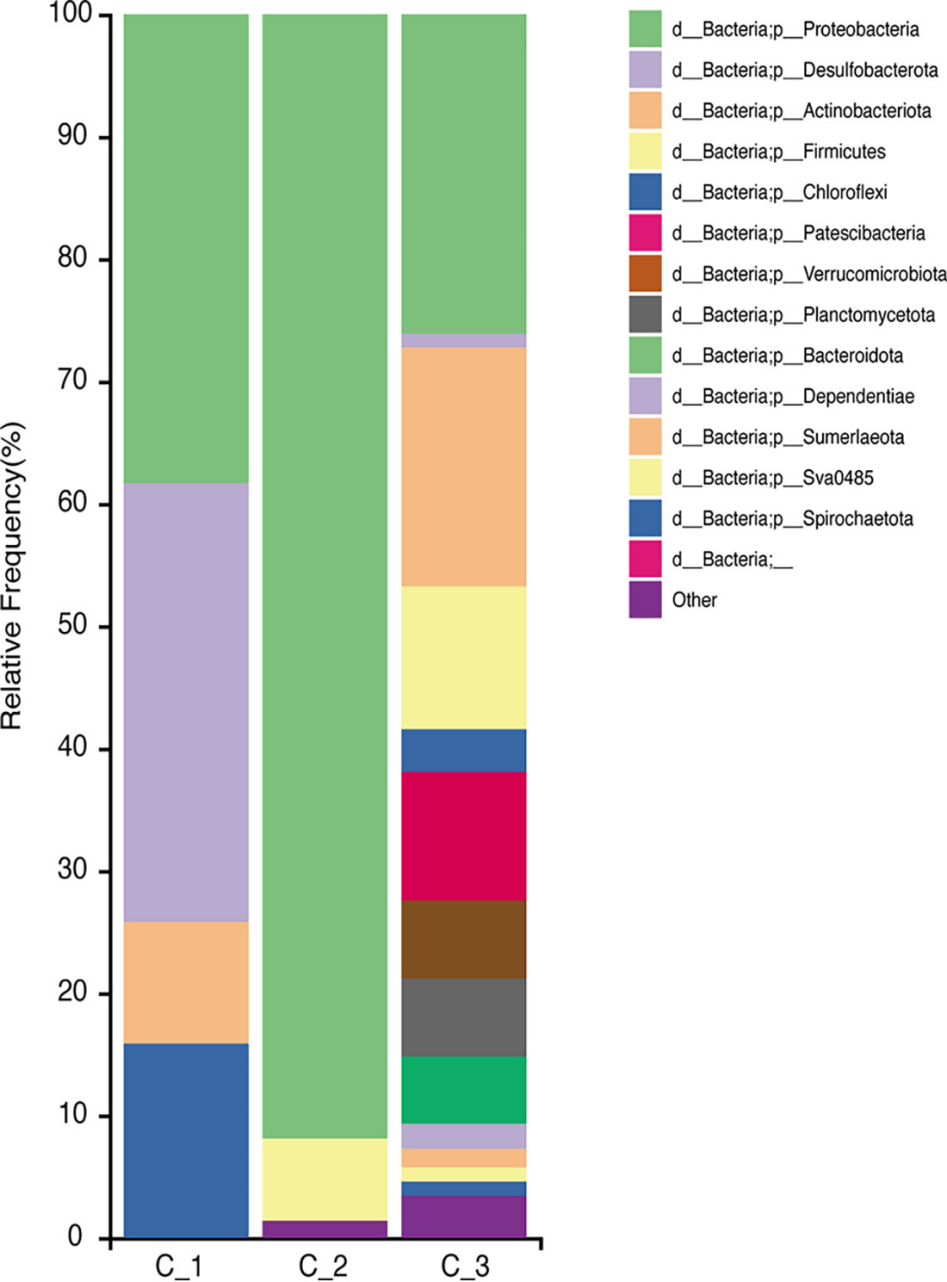
Bar chart representing the taxonomic composition of the gut microbiota of Chimaera phantasma. The relative abundances of the taxa are shown at the phylum level. The “Other” category includes taxa with relative abundance of <1%. C_1 and C_2, females; C_3, male.

The three samples contained a total of 123 OTUs. *Proteobacteria* constituted >90% of the microbiota in sample C_2 (Fig. 1), comprising 89.2% *Photobacterium* and 2.3% *Shewanella* species. *Photobacterium* species are luminous, halophilic, Gram-negative species that are present in the gut of deep-sea fish (12). *Shewanella* species are barotropic, luminous, Gram-negative species that are found in oceans (13–15). *Proteobacteria* constituted 38.3% (4 species) of the species in sample C_1 and 26.1% (22 species) of those in sample C_3. Other taxa with occupancy rates of ≥10% were *Actinobacteria* (19.5%), *Firmicutes* (11.7%), and *Patescibacteria* (10.5%) in sample C_1 and *Desulfobacterota* (35.9%) and *Chloroflexi* (15.9%) in sample C_3.

### Data availability.

The 16S rRNA gene amplicon sequence data have been deposited in the DDBJ Sequence Read Archive (DRA) under the accession numbers DRR413612, DRR413613, and DRR413614.
